# MiR-155 inhibits cell migration of human cardiomyocyte progenitor cells (hCMPCs) *via* targeting of MMP-16

**DOI:** 10.1111/j.1582-4934.2012.01551.x

**Published:** 2012-09-26

**Authors:** Jia Liu, Alain Mil, Eissa N E Aguor, Sailay Siddiqi, Krijn Vrijsen, Sridevi Jaksani, Corina Metz, Jiajun Zhao, Gustav J Strijkers, Pieter A Doevendans, Joost P G Sluijter

**Affiliations:** a Department of Endocrinology, Provincial Hospital affiliated to Shandong UniversityJinan, China; b Department of Cardiology, University Medical CenterUtrecht, the Netherlands; c Interuniversity Cardiology Institute of the Netherlands (ICIN)Utrecht, the Netherlands; d Biomedical NMR Department of Biomedical Engineering, Eindhoven University of Technologythe Netherlands

**Keywords:** cardiomyocyte progenitor cells, microRNA, cell migration, matrix metalloproteinases

## Abstract

Undesired cell migration after targeted cell transplantation potentially limits beneficial effects for cardiac regeneration. MicroRNAs are known to be involved in several cellular processes, including cell migration. Here, we attempt to reduce human cardiomyocyte progenitor cell (hCMPC) migration *via* increasing microRNA-155 (miR-155) levels, and investigate the underlying mechanism. Human cardiomyocyte progenitor cells (hCMPCs) were transfected with pre-miR-155, anti-miR-155 or control-miR (ctrl-miR), followed by scratch- and transwell- assays. These functional assays displayed that miR-155 over-expression efficiently inhibited cell migration by 38 ± 3.6% and 59 ± 3.7% respectively. Conditioned medium from miR-155 transfected cells was collected and zymography analysis showed a significant decrease in MMP-2 and MMP-9 activities. The predicted 3′-UTR of MMP-16, an activator of MMP-2 and -9, was cloned into the pMIR-REPORT vector and luciferase assays were performed. Introduction of miR-155 significantly reduced luciferase activity which could be abolished by cotransfection with anti-miR-155 or target site mutagenesis. By using MMP-16 siRNA to reduce MMP-16 levels or by using an MMP-16 blocking antibody, hCMPC migration could be blocked as well. By directly targeting MMP-16, miR-155 efficiently inhibits cell migration *via* a reduction in MMP-2 and -9 activities. Our study shows that miR-155 might be used to improve local retention of hCMPCs after intramyocardial delivery.

## Introduction

Undesired cell migration and sequential low cell retention post-intramyocardial injection potentially limits the therapeutic effect of cell therapy for cardiac regeneration [1]. With the progresses in catheter development for targeted cell injection, specific cell delivery can be more successfully achieved and especially myocardium in need could receive more implanted cells [2–5]. However, direct cellular retention is limited after delivery because of myocardial contraction and catheter misplacement. In addition, inhibiting undesired cellular migration in the heart or to other organs post-transplantation might enhance the efficiency of cellular therapy as a result of improved retention of delivered cell in the myocardium in need [5].

During recent years, small RNA molecules, *i.e*. microRNAs (miRNAs) – a class of 21–25 nucleotide long non-coding RNAs– have been found to keep tight control of various processes by gene or gene cluster silencing. Several lines of evidence showed that miRNAs are involved in cellular migration and could efficiently modulate cell motility by down-regulation of target genes [6–8]. Examples include a brain-specific miRNA, miR-9, that targets stathmin and thereby inhibits human embryonic neural progenitor cell migration in a stroke model in adult mouse brains [9], and miR-10a which promotes human pancreatic cell migration by suppressing HOX genes, HOX1 and HOXB3, in zebrafish embryos [10]. These data indicate the possibility for modulating miRNAs, limiting cell migration and thereby keeping maximum retention of cells within the myocardium after targeted local administration.

Previously, we have shown that miR-155 significantly attenuated necrotic cell death in human cardiomyocyte progenitor cells (hCMPC) and suggested the possibility of promoting cell survival upon injection, thereby increasing the efficiency of cell-based therapy [11]. Here, we report that miR-155 can also block hCMPC migration by directly targeting matrix metalloproteinase (MMP)-16, an activator of MMP-2 and-9, thereby reducing potential off-target migration and potentially leading to improved targeted cell delivery.

## Methods

### Cell culture and expansion

The hCMPCs were isolated and expanded as previously reported [12]. In short, human foetal heart tissue was collected after elective abortion and digested by collagenase to obtain a single cell suspension. hCMPCs were isolated by magnetic cell sorting (MACS, 130-090-312; Miltenyi Biotech, Bergisch Gladbach, Germany) using a mouse anti-Sca-1 antibody (130-091-176; Miltenyi Biotech) and cultured as described [13]. Three individual cell lines were characterized and used for all experiments (CMPC-1, CMPC-2, CMPC-3). Standard informed consent procedures and prior approval of the ethics committee of the University Medical Center Utrecht were obtained.

### Small RNA transfection

The pre-miR™ precursor molecules for miR-155 (PM 12601) (pre-miR-155), anti-miR™ inhibitor for miR-155 (AM 12601) (anti-miR-155) and a negative control-miR (AM 17110) (ctrl-miR) were obtained from Ambion (Austin, TX, USA). Stealth RNAi for MMP-16 (set of 3; low GC, HSS106647, HSS181141 and HSS181142), and Stealth RNAi negative control (Low GC, 12935200) were purchased from Invitrogen (Carlsbad, CA, USA). hCMPCs were transfected with 0, 3, 30 or 100 nM of appropriate miRNAs, as reported before [11] or 100 nM of siRNA with Lipofectamine™ Transfection Agent (Invitrogen), according to the manufacturer's guidelines. Transfection efficiency of miRNAs or siRNAs was confirmed by RT-PCR and western blot.

### qRT-PCR for miRNA expression

Total RNA was isolated with Tripure reagent™ (Roche Applied Science). 3.3 ng DNAse-free total RNA was used for reverse transcription (Taqman® MicroRNA Reverse Transcriptase Kit, Applied Biosystems, Foster City, CA, USA) followed by Taqman® MicroRNA Assays for quantification of miR-155 (4373124; Applied Biosystems) and RNU19 (4373378; Applied Biosystems) control transcripts. Amplification and detection of specific PCR products was performed in a MyIQ single-colour real-time PCR system (Bio-Rad, Hercules, CA, USA). miR-155 expression levels were normalized for RNU19 and presented as fold induction (2^−ΔΔCt^).

Matrix metalloproteinase-16 mRNA expression was determined by using the SuperScript First-Strand Synthesis System (170-8890; Bio-Rad) and qRT-PCR amplification was detected in a MyIQ single-colour real-time PCR system by using iQ™ SYBR® Green Supermix (170-8884; Bio-Rad) and specific primers. (Forward: GGACAGAAATGGCAGCACAAGC, reverse: CATCAAAGGCACGGCGAATAGC). PCR product was loaded on a PAGE-GEL to visualize and determine specificity of the amplified products.

### Zymography

Cells were transfected with different miRNAs as described above and refreshed with serum free medium the day after transfection. Conditioned medium was collected for 48 hrs and loaded on a SDS-PAGE supplemented with gelatin substrate [14, 15], followed by incubation with Brij solution (50 mM Tris, 10 mM CaCl_2_, 0.05% Brij-35 solution) at 37°C O/N and stained with Coomassie blue. The intensities of digested bands were detected by using ChemiDoc XRS system (Bio-Rad).

### Scratch assay

Cells were transfected with different miRNAs as described above. When cells reached confluency (24–48 hrs) after transfection, a scratch wound was made and hCMPC migration was monitored subsequently for 6–8 hrs. To quantify hCMPC migration, pictures were made immediately after the generation of the scratch and at the termination of the experiment. Photoshop and Image J software were used to calculate the area of wound closure.

### Transwell assay

Cells were transfected with different small RNAs 48 hrs before transwell assay. 5 × 10^4^ transfected cells were resuspended in basic DMEM culture medium, supplemented with 0.5% FBS, and added to the upper chamber of a transwell system (8 μm pore size, Corning 3422). Basic culture medium, supplemented with 10% FBS and 10 ng/ml VEGF, was added to the lower chamber. Cells were allowed to migrate for 6–8 hrs before membranes were fixed. The non-migrated cells from the upper well were removed by a cotton wrap, and migrated cells were stained with 0.2% crystal violet, followed by quantification using Image J.

### Target site cloning

Of the 4.2 kb long MMP-16 3′UTR, the last 1.4 kb, containing the two putative binding sites for miR-155, was cloned into the pMIR-REPORT vector (Ambion, 5795). For this, we used the following primers with restriction sites (Italic) for subcloning: mmp16 3′UTR forward with SpeI site 5′-*GACTAGTC*GGGCCTTGATGTCAAGAAAA-3′ and mmp16 3′UTR reverse with SacI site 5′-*CGAGCTCG*CCCACAGAGAGGGAATGAAA-3′.

The obtained plasmids were isolated, followed by sequencing (Hubrecht Lab, the Netherlands) to confirm sequences of the inserted MMP-16 3′-UTR and vector. Mutational cloning in the seed region of predictive sites was performed with QuikChange® Site-Directed Mutagenesis Kit (200518; Stratagene, La Jolla, CA, USA), according to manufacturer's guidelines. To achieve the two mutations in the putative seed regions, we used the following primers (mutated nucleotides are in bold): mutation site 1 forward: 5′-GGGCTAGAAAATAATCATAG**G**A**G**TAAGAAGGAAGGAGTCTATC-3′, mutation site 1 reverse: 5′-GATAGACTCCTTCCTTCTTA**C**T**C**CTATGATTATTTTCTAGCCC-3′, mutation site 2 forward: 5′-GAGCTAAATTAGACAG**G**A**G**TATGTGATACTAGCAAAGACAACTTC-3′ and mutation site 2 reverse: 5′-GAAGTTGTCTTTGCTAGTATCACATA**C**T**C**CTGTCTAATTTAGCTC-3′.

### Luciferase experiments

In a 24-well plate, HEK 293 cells were transfected with 200 ng pMIR-REPORT vector, containing the MMP-16 3′UTR, or seed-mutated vectors, in combination with 100 nM of different miRNAs. A total of 200 ng CMV-β-galactosidase plasmid was cotransfected as an internal transfection control. Cells were lysed 48 hrs after transfection and luciferase and β-galactosidase activities were measured, as described [14].

### Western blotting

Forty-eight hours after miRNAs or siRNA transfection, total protein lysate was extracted using RIPA lysis buffer (50 mM Tris/HCl pH7.5, 0.1% SDS, 1% Triton-X 100, 0.5–1% sodium deoxycholate, 150 mM NaCl, protease inhibitors (04693159001; Roche, Penzberg, Germany). Samples were loaded on a NuPAGE® Novex® Bis-Tris Gel (NP0336; Invitrogen) with 10x reducing reagent (NP004; Invitrogen). Gels were transferred to a PVDF membrane (32-10413096; Schleicher & Schuell, Dassel, Germany), subsequently incubated with MMP-16 antibody (rabbit polyclonal, 73877, 40 ng/ml; Abcam, Cambridge, UK) and goat-anti-rabbit IgG secondary antibody (P0448, 1:2000; Dako, Glostrup, Denmark). Beta-tubulin (rabbit polyclonal, 2146, 1:2000; Cell Signaling, Danvers, MA, USA) was used as loading control. Signals were detected after exposure to enhanced chemiluminescence substrate (Amersham) by using the ChemiDoc XRS system (Bio-Rad).

### Immunohistochemistry

The hCMPCs were seeded in chamber slides O/N and fixed with 4%PFA for 10 min. at RT. Cells were washed with 0.2% Triton in PBS for 5 min. at RT, blocked with 2% PBSA for 60 min. and incubated with rabbit-anti-human MMP-16 (ab73877; Abcam) or isotype (rabbit IgG) in 2% PBSA O/N at 4°C. MMP-16 was visualized by using goat-anti-rabbit Alexa555 for 60 min. at RT in the dark and imaged by using a Zeiss LSM 510 Meta confocal microscope.

### MMP-16 blocking antibody

The hCMPCs were seeded in 24-well plates at 50% confluency in normal culture medium. The next day, a scratch wound was created and the medium was refreshed with 1% FBS culture medium, which contained 20 μg/ml MMP-16 (73877; Abcam) or IgG isotype control antibody. After 6–8 hrs, cell recovery was monitored and analysed by using Photoshop software.

### Statistics

Data are presented as mean ± S.E.M. of at least three independent experiments and were compared using the two-tailed paired Student's *t*-test. A difference with a *P* < 0.05 was considered to be statistically significant.

## Results

### Introducing miR-155 inhibits cell migration

**Fig 1:**
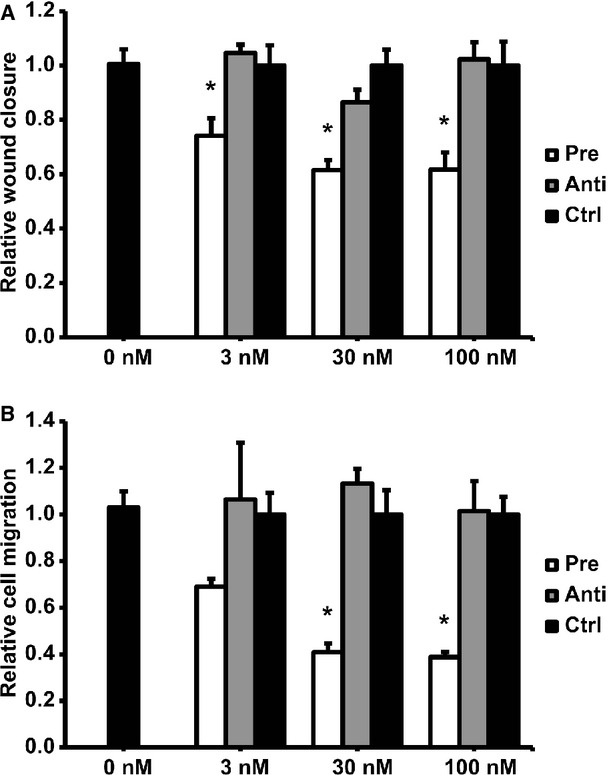
Introducing miR-155 in hCMPCs reduced cell migration in scratch (A) and transwell assays (B). Cells were transfected with different concentrations (0, 3, 30, 100 nM) of pre-miR-155 (pre), anti-miR-155 (anti) and ctrl-miR (ctrl), normalized to non-transfected cells. Data are presented as mean ± S.E.M., *N* = 3 and *P < 0.05.

As increased miR-155 levels could improve cell survival [13] and thereby potentially increase cell retention, we studied if increasing miR-155 levels might contribute to improved cell retention *via* other mechanisms, thereby exploring whether miR-155 over-expression could attenuate hCMPC cell migration. For this, we performed a scratch wound assay and monitored wound closure for 6–8 hrs. Overexpressing miR-155 was achieved and confirmed by qRT-PCR as previously reported [11]. We observed that increasing levels of miR-155 inhibited cell migration and showed that 30 nM pre-miR-155 reduced migration by 38 ± 3.6% compared to ctrl-miR (Fig. 1A, *P* < 0.05). Furthermore, to exclude an effect of hCMPC proliferation, we performed a transwell migration assay. Introducing 30 nM pre-miR-155 decreased migration over a membrane with 59 ± 3.7%, as compared to the ctrl-miR group (Fig. 1B, *P* < 0.05). These combined data suggest that miR-155 is effective in blocking hCMPC cell migration.

### MiR-155 reduces MMP-2 and -9 activity levels

**Fig 2:**
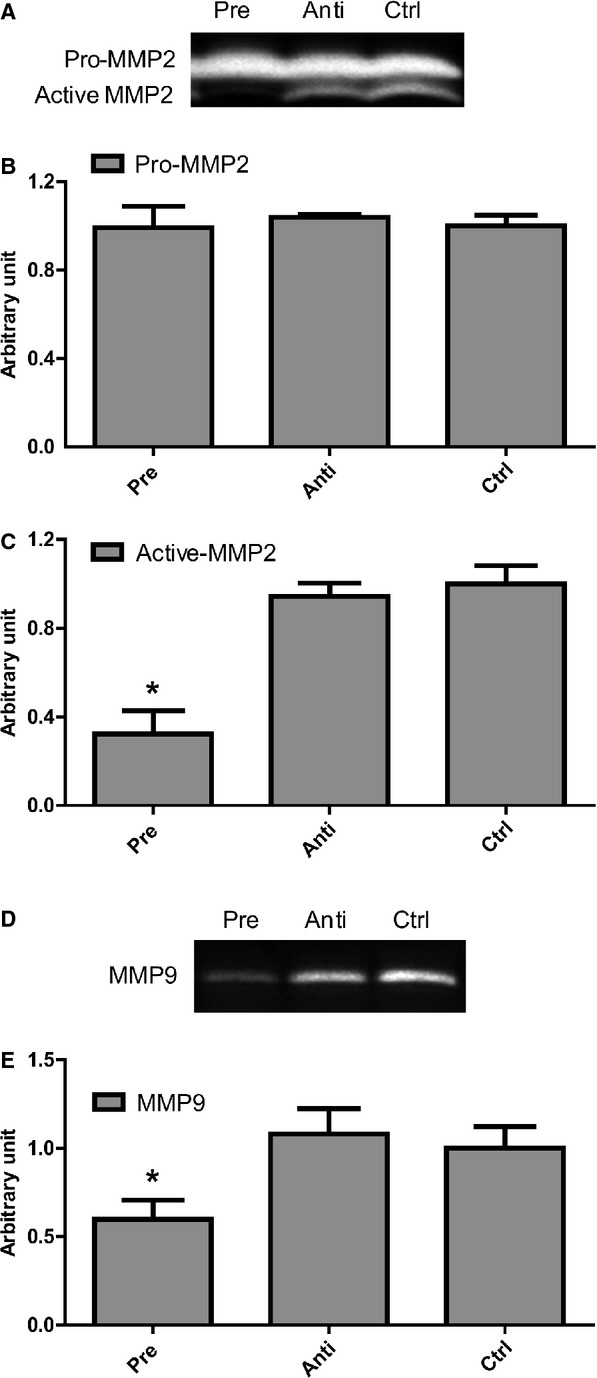
Introducing 30 nM pre-miR-155 in hCMPCs decreased matrix metalloproteinase (MMP) activity levels as detected by zymography. Visualization (A) and quantification of pro- (B) and active-MMP-2 (C) activity. Visualization (D) and quantification (E) of MMP-9 activity levels. Data are presented as mean ± S.E.M., *N* = 3 and *P < 0.05.

We have observed before that hCMPCs are able to produce MMP-2 and -9 [16], important proteases that allow matrix turnover and cell migration. We tested secreted MMP-2 and -9 levels from hCMPCs upon transfection of different miRNAs. Overexpressing miR-155 decreased active-MMP-2 and -9 levels by 68% (Fig. 2A and C, *P* < 0.05) and 49% (Fig. 2D and E, *P* < 0.05) respectively. Interestingly, pro-MMP-2 levels were not affected (Fig. 2A and B), indicating that miR-155 limits cell migration by inhibiting MMP-2 and -9 activation, but not by affecting their expression. This was confirmed by unchanged MMP-2 and -9 mRNA levels (Suppl Fig. 1). As miRNAs cannot block protease activity and because MMP-2 and -9 are not predicted to be targets of miR-155, we explored additional potential mechanisms.

### MiR-155 directly targets MMP-16 (MT3-MMP), an activator of MMPs

**Fig 3:**
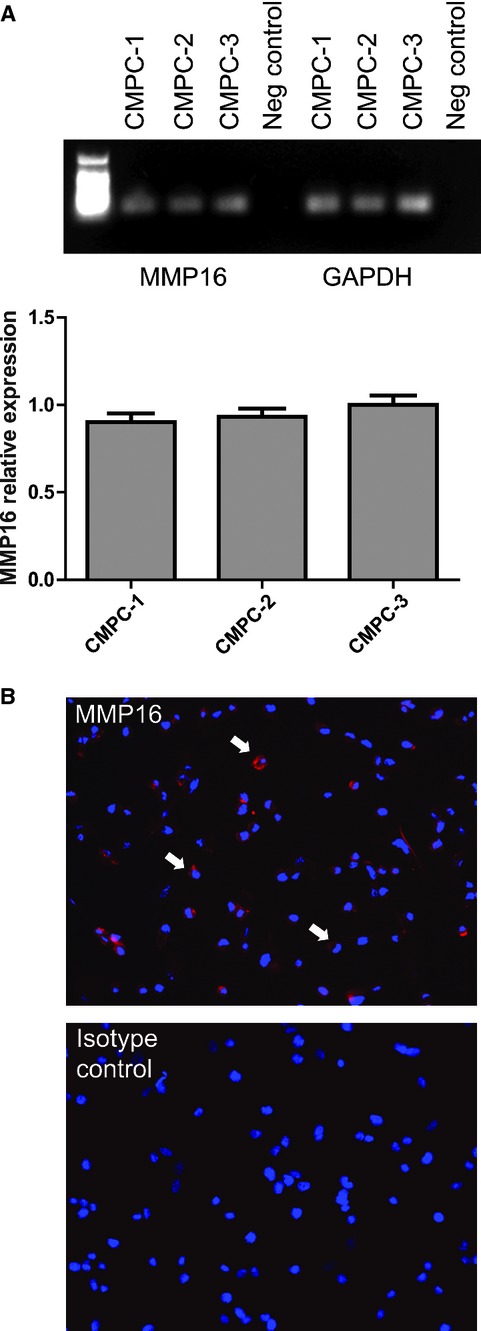
Matrix metalloproteinase-16 (MMP-16) is expressed in hCMPCs as indicated by (A) MMP-16 gene expression in different hCMPC cell lines, and (B) immunofluorescent analysis for MMP-16 in hCMPCs. (MMP-16 expression in red, positive cells are indicated by arrows).

MiR-155 is predicted to target MMP-16 (MT3-MMP, membrane type3 MMP) (http://www.microRNA.org), which is a potential activator of MMP-2 and -9 [17, 18]. We therefore examined whether miR-155 could directly target MMP-16. As MMP-16 expression was not detected before in hCMPCs, we explored and confirmed that MMP-16 is expressed in different primary cell lines of hCMPC as indicated by gene expression and immunohistochemistry (Fig. 3A and B).

**Fig 4:**
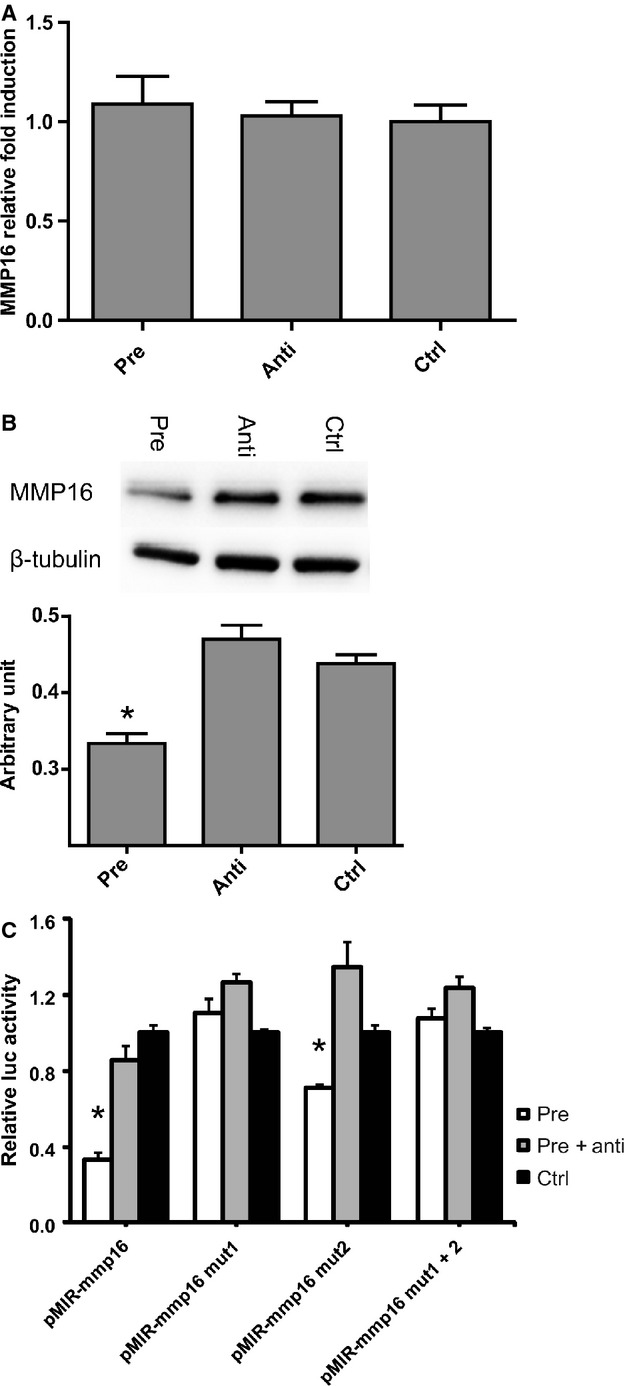
MMP-16 expression in miRNAs transfected hCMPCs, on mRNA level (A) and on protein level (B), as demonstrated by western blotting, including densitometric quantification. Luciferase activity of either pMIR-REPORT vector, containing a part of the 3′-UTR of MMP-16 (pMIR MMP-16) or containing the MMP-16 3′-UTR with seed-mutated targets sites 1, 2 or both (C). Data are presented as mean ± S.E.M., *N* = 4 and *P < 0.05, pre = pre-miR-155, anti = anti-miR-155, ctrl = control-miR).

Upon pre-miR-155 transfection, MMP-16 mRNA expression levels did not change in hCMPCs (Fig. 4A), however, a robust down-regulation of MMP-16 protein expression could be observed (Fig. 4B, *P* < 0.05 and Fig. S2). This suggests that MMP-16 is a direct target of miR-155. To confirm this, we cloned 1.4 kb of the 3′-UTR of MMP16 into a luciferase reporter vector and could observe a significant reduction in luciferase expression after cotransfecting miR-155 (Fig. 4C). This inhibition could be completely abolished by cotransfection with anti-miR-155, indicating that miR-155 directly down-tunes MMP-16 production. This was further confirmed by generation of single mutations in the two putative target sites of MMP-16. We could observe that the decrease in luciferase activity was prevented (Fig. 4C), and that site 1 is probably most powerful in regulating MMP-16 expression.

### MMP-16 knockdown efficiently inhibits cell migration

**Fig 5:**
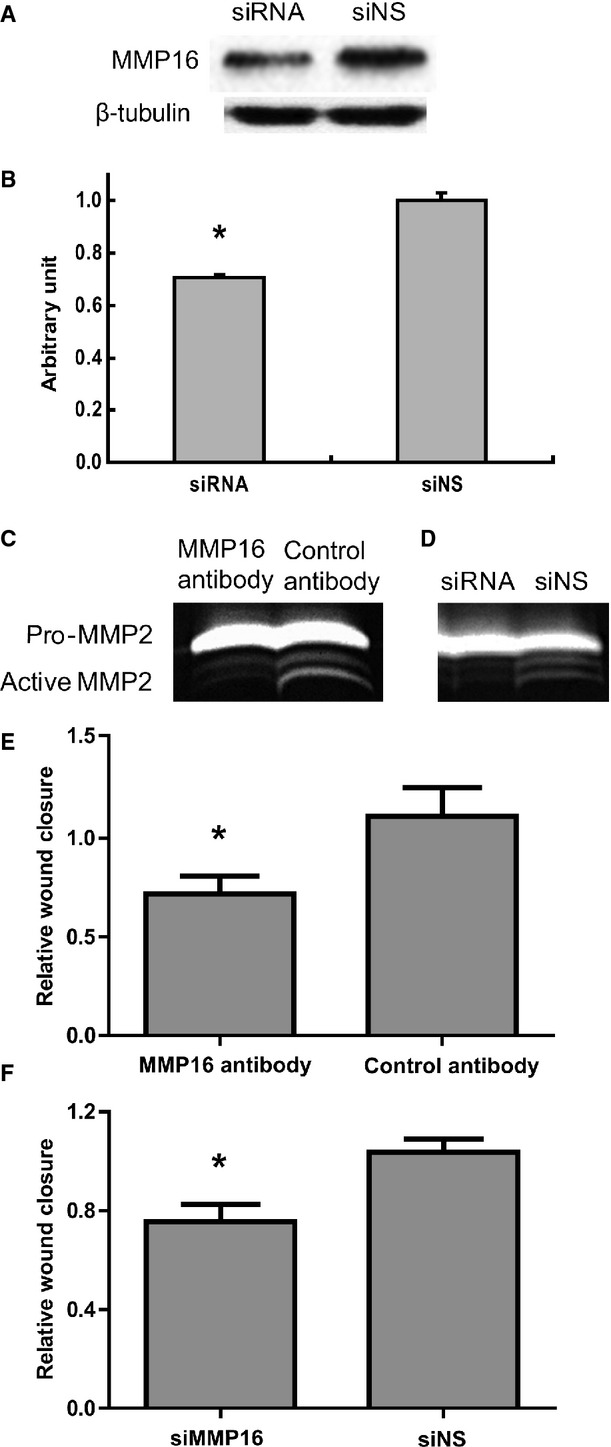
By using siRNA for MMP-16 we could reduce MMP-16 protein levels (A), as quantified in (B). Both a blocking antibody for MMP-16 (C) as the siMMP-16 (D) reduced MMP-2 activation, as visualized by zymography (C, D). Both approaches resulted in a reduction in cell migration in a scratch assay as shown for the specific MMP-16 antibody (E) or by using siRNA knockdown of MMP-16 (F). Data are presented as mean ± S.E.M., *N* = 3 and *P < 0.05.

To test the functional relevance of MMP-16 in the observed reduction in MMP activities and migration, we used an MMP-16 blocking antibody or knocked down MMP-16 expression by siRNA treatment. As shown, RNAi efficiently repressed MMP-16 expression in hCMPCs (Fig. 5A and B). Blocking MMP-16 with blocking antibody (Fig. 5C) or RNAi could also efficiently inhibit MMP-2 activity (Fig. 5D). Scratch assays showed that blocking MMP-16 by MMP-16 antibody treatment or RNAi diminished cell migration to a similar extent as miR-155 over-expression (Fig. 5E and F). Our combined results show that miR-155 directly targets MMP-16 and thereby consequentially reduces MMP-2 and MMP-9 activity levels, leading to efficient inhibition of cell migration.

## Discussion

Short-term cell retention is a pre-requisite for long-term cell engraftment upon cell transplantation therapy, thereby functionally enhancing cardiac repair. Good survival and limited undesired cell migration could enhance cell retention as was previously indicated [5]. Co-administration of cardiac progenitor cells and IGF-1 promoted better cell engraftment and thereby facilitated cardiac regeneration [19]. Much effort has been put into improving cell survival and engraftment after transplantation for cell-based therapy, like pre-conditioning MSCs with stromal-derived factor1 alpha (SDF-1) [20] or over-expression of Akt, which protected MSCs from death after transplantation *via* a paracrine mechanism [21]. Ischaemic pre-conditioning augmented miR-210 expression and thereby protected MSC survival by targeting caspase8 associated protein [22]. We have also reported previously that miR-155 over-expression efficiently inhibited necrotic cell death *via* targeting RIP1 (receptor interacting protein1), providing a potential novel approach to improve cell engraftment [11].

In addition to increased cell survival, limiting off-target migration will also potentially lead to a better cell retention. Currently, therapeutic cells can be injected accurately into the area of interest with the guidance of *e.g*. the NOGA system. This system incorporates a cardiac catheter device and a three dimensional electromechanical mapping system, and thereby can define the viable, hibernating and infarcted myocardium [1]. We previously observed that hCMPC transplantation repressed left ventricular remodelling and resulted in better cardiac performance. Upon injection, engrafted cells were not only confined to the site of injection, but could be detected in the remote regions, indicating that cells are able to migrate away from the in-need myocardium [23]. This highlights the necessity of limiting post-delivery cell migration in the pre-defined myocardium.

MiRNAs are known to be involved in various cellular processes, including cell migration [24–26]. Although most studies showing that miRNAs interfere with migration are focused on tumour cell invasion [27–29], several lines of evidence showed that miRNAs play important roles in non-tumour cell migration as well. Delaloy *et al*. reported that brain-specific miR-9 is turned on in human neural progenitors and coordinates cell proliferation and migration *via* targeting stathmin, a microtubule stability inhibitor [9]. Sarkar *et al*. showed that miR-21 enhanced hypoxia-mediated pulmonary artery smooth muscle cell migration by targeting programmed cell death protein 4 (PDCD4), Sprouty 2 (SPRY2) and Peroxisome-Proliferator-Activated-Receptor Alpha (PPARa-alpha) [25].

In this study, we investigated the potential use of increasing miR-155 levels in hCMPCs to influence cell migration. Functional assays demonstrated that miR-155 efficiently blocked cell motility mediated by reduced MMP-2 and -9 activities. The role of miR-155 in cellular migration seems rather cell type and context specific as some reports have shown both promoting as well as inhibiting roles for miR-155 in different cell sources [30, 31]. Furthermore, we found that miR-155 directly down-regulated MMP-16, an activator of MMP-2 and -9, and that inhibition of MMP-16 by a blocking antibody or *via* siRNA knockdown reduced hCMPC migration to a similar extent as miR-155 over-expression. MMP-16 is a membrane anchored MMP that is able to activate other MMPs, growth factors and receptors, and thereby facilitates a local cellular mechanism for migration. Our results confirm the functional relevance of MMP-16 in the down-regulation of hCMPC cell migration. This is in concordance with a previous report which showed that decreasing MMP-16 levels efficiently limited cell migration of glioma cells [32].

Although increased expression of miR-155 could efficiently modulate cell migration, we did not observe a block in migration when endogenous levels of miR-155 in hCMPCs were blocked. This suggests that the biological endogenous role of miR-155 in hCMPCs may not be migratory. However, as pre-miR-155 transfection only transiently increased miR-155 levels, pre-conditioning hCMPCs with miR-155 provides a novel opportunity to improve cell survival and reduce migration after transplantation from a therapeutic point of view.

In conclusion, increasing miR-155 levels can inhibit hCMPC migration *via* direct targeting of MMP-16 *in vitro*. In combination with the cytoprotective effect [13], miR-155 might provide a novel and promising approach to augment the therapeutic effect of hCMPCs transplantation. Additional proof of improved targeted delivery mediated by miR-155 *in vivo* is needed for further evaluation of its possible role in cell transplantation therapy.

## Supporting information

**Figure S1** MMP-2 mRNA expression in different miRNAs transfected cells. Data are presented as mean ± S.E.M., *N* = 3.

**Figure S2** Western blot of MMP-16 expression upon transfection of different concentrations of pre-miR-155 (3, 30 and 30 nM) as compared to control and anti-miR-155 transfected hCMPCs.
